# Pulmonary Involvement during the Ebola Virus Disease

**DOI:** 10.3390/v11090780

**Published:** 2019-08-24

**Authors:** Eleonora Lalle, Mirella Biava, Emanuele Nicastri, Francesca Colavita, Antonino Di Caro, Francesco Vairo, Simone Lanini, Concetta Castilletti, Martin Langer, Alimuddin Zumla, Gary Kobinger, Maria R. Capobianchi, Giuseppe Ippolito

**Affiliations:** 1 National Institute for Infectious Diseases ‘Lazzaro Spallanzani’ IRCCS, 00149 Rome, Italy; 2 International Public Health Crisis Group, 00149 Rome, Italy; 3 EMERGENCY Onlus NGO, Via Santa Croce 19, 20122 Milan, Italy; 4 International Public Health Crisis Group, London WC1E 6BT, UK; 5 Division of Infection and Immunity, National Institute for Health Research Biomedical Research Centre at University College London Hospitals NHS Foundation Trust, London WC1E 6BT, UK; 6 International Public Health Crisis Group, Quebec City, PQ G1V 0A6, Canada; 7 Department of Medical Microbiology and Infectious Diseases, University of Manitoba, Winnipeg, MB R3E 0J9, Canada; 8 Département de microbiologie-infectiologie et d’immunologie, Université Laval, Québec, PQ G1V 0A6, Canada

**Keywords:** Ebola virus, lung pathogenesis, respiratory disease, Ebola virus disease

## Abstract

Filoviruses have become a worldwide public health concern, especially during the 2013–2016 Western Africa Ebola virus disease (EVD) outbreak—the largest outbreak, both by number of cases and geographical extension, recorded so far in medical history. EVD is associated with pathologies in several organs, including the liver, kidney, and lung. During the 2013–2016 Western Africa outbreak, Ebola virus (EBOV) was detected in the lung of infected patients suggesting a role in lung pathogenesis. However, little is known about lung pathogenesis and the controversial issue of aerosol transmission in EVD. This review highlights the pulmonary involvement in EVD, with a special focus on the new data emerging from the 2013–2016 Ebola outbreak.

## 1. Introduction

Ebolavirus is part of the Filoviridae family, which consists of three genera: *Marbugvirus, Cuevavirus*, and *Ebolavirus*. There are currently six known, genetically distinct, species of *Ebolavirus*—*Ebola virus* (EBOV), *Sudan Ebolaviurs* (SUDV), *Tai Forest Ebolavirus* (TAFV), *Bundibugyo Ebolavirus* (BDBV), *Reston Ebolavirus* (RESTV), and *Bombali Ebolavirus* (BOMV) [1,2]. No virus has triggered fear in the general population more than the filovirus Ebolavirus [3]. EBOV is categorized among the deadliest viruses, with mortality rates up to 90%. The zoonotic origin of outbreaks are often the result of transmission from primates, although the suspected natural reservoir for EBOV, bats, is still being questioned. Since it was first identified in 1976 in Zaire (the actual Democratic Republic of Congo), 27 confirmed outbreaks, mainly in the central part of Africa, have occurred, and each outbreak was accompanied by high case fatality rates up to 88%, including the new declared outbreak ongoing in the North Kivu Province of the Democratic Republic of the Congo [4,5,6]. The 2013–2016 Ebola outbreak is the largest (both by number of cases and geographical extension) ebolavirus outbreak ever reported, resulting in 28,610 cases and 11,308 deaths, with fatality rates of 70% in Guinea and Sierra Leone and 41% in Liberia [5]. The number of cases in this single outbreak is far greater than the total number of all cases and deaths of the past outbreaks over the last 40 years. The reasons of such an extended outbreak are linked to societal factors (poverty, urbanization, population migration patterns, and changes of socio-economic conditions), together with the concomitant invasion of animal habitats, climate change, and deforestation [4]. In fact, the emergence and re-emergence of such viruses in Africa or their potential introduction into new countries have usually been related to the mobility and international transport of infected animals or animal products, thus making ebolavirus and filoviruses a worldwide public health concern [7]. Moreover, despite almost 40 years of research, filovirus transmission remains incompletely understood. In humans, EBOV has been found in a variety of body fluids, including blood, stool, breast milk, semen, urine, and saliva [8]. There are multiple routes of transmission for EBOV. However, information about transmission in humans is incomplete, and defining the modes of transmission would greatly increase the ability of public health structures to limit the disease, as well as enable health care workers to avoid any unnecessary risk. So far, our understanding of EBOV transmission in humans mainly relies on epidemiological observations and contact with body fluids from EBOV-positive patients remain the most likely route of transmission. Notably, the number of past outbreaks and associated epidemiological studies carefully examining transmission patterns are small. 

Ebola Virus Disease (EVD) is commonly associated with multiple organ systems, including the liver, renal organs, and lungs [9]. So far, little is known about the involvement of the respiratory tract and EBOV pathogenesis in the lung. However, little evidence in filovirus animal outbreaks and animal studies highlights the involvement of the lungs and the respiratory tract in filovirus pathology. Over the years, there has been an increasing concern regarding the possible involvement of the lung in EBOV infection. This concern further increased during the 2013–2016 EBOV outbreak, which offered evidence of viral shedding in the lung, leading to a risk of aerosol transmission [10]. The aim of this review is to highlight the pulmonary involvement in EVD, with a special focus on the new data emerging from the 2013–2016 Ebola outbreak.

## 2. Host Defense Mechanisms in the Respiratory Tract during EBOV Infection

**Figure 1:**
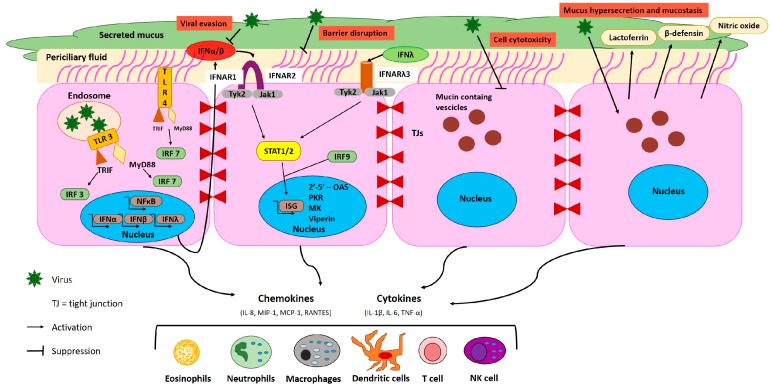
Direct and indirect effects of viral infections of the airway epithelium. Upon entrance into the cell, viruses are recognized by the Toll-like receptor (TLR) on either cell membrane or in endosomes. TLRs activate interferon regulatory factors (IRFs) leading to IFN-α and IFN-β release via the Toll/IL-1 receptor domain-containing adaptor (TRIF). TLR3 stimulates IRF-7 and NF-κB via MyD88 activation, leading to the release of proinflammatory cytokines and the production of IFN-α, -β, and -λ, respectively. Secretion of proinflammatory cytokines and chemokines activate the immune system, through recruitment of eosinophils, neutrophils, macrophages, dendritic cells, T cells, and NK cells. Most respiratory viruses have developed strategies to escape antiviral defense, mainly by interfering with the IFN system or by affecting the epithelium barrier, with the consequence of a loss of integrity and protection. Furthermore, respiratory viruses can perturb (skewed or exaggerated) inflammatory responses and production of soluble mediators.

The lung is a vulnerable organ along with the skin and the gut; it is the interface between the sterile body sanctuary and the external environment. Besides its role in maintaining the conduit for air to and from the alveoli, the airway epithelium of the respiratory system is central to the defense of the lung against pathogens. Through the combined function of ciliated epithelial and secretory cells, efficient mucociliary clearance is maintained, along with a variety of other host defense mechanisms [11]. Airway epithelial cells regulate both innate and adaptive immunity through production of functional molecules and physical interactions with cells of the immune system [12]. Despite these defense mechanisms, viruses have found ways to evade the immune system, resulting in severe respiratory disease. Lung pathogenesis is a combination of direct and indirect processes involving virus and/or host factors. Virus-related factors (Figure 1) include receptor specificity and the ability to induce cell damage (direct cytopathic effect) and/or cell apoptosis, whereas host-related factors mostly include the inflammatory response and the activation of the immune system, combined with host-risk factors (chronic respiratory diseases, smokers, atopy of the airway epithelium, etc). 

EBOV infection is acquired through direct contact with bodily fluids. The virus enters blood circulation through breaks in the skin and mucosa and spread to different organs, causing systemic manifestation of cardiovascular, coagulation, or inflammatory disturbances [10]. The terminal stages of EVD usually involve massive tissue injury and hemorrhage, resulting in multiorgan failure and shock, the main cause of exitus in EVD patients [13]. During EVD, respiratory symptoms such as chest pain, shortness of breath, cough, and nasal discharge are signals of the multisystem involvement, but, so far, lung damage has not been directly linked to EBOV replication in the respiratory tract. However, new evidences collected during the recent 2013–2016 Ebola outbreak hypothesized shedding of the virus in the lung and identified viral replication markers in sputum samples collected from EBOV infected patients [14]. On the other hand, the high virulence of EBOV is attributed in large part to the ability of this virus to interfere with the host immune response, and the high degree of variation in lung pathogenesis is usually linked to indirect damage due to endothelial and epithelial inflammation and the hyper-activation of the immune system subsequent to EBOV infection. In fact, viral direct damages are always associated with indirect damages, caused by inflammatory and immune reactions elicited by the viruses through the activation of soluble mediators (cytokines and chemokines) as part of the immune response (Figure 1). The acute inflammation process is characterized by increasing blood flow, which enables plasma and leukocytes to reach extra-vascular sites of injury. Even though inflammation may be often restored, in EVD, severe inflammation is associated with a cytokine storm and more serious pathological changes are observed. For instance, EBOV in vitro infection of monocytes and macrophages triggers the robust expression of inflammatory mediators, including IL-1β, IL-6, IL-8, MIP-1a, MIP-1β, MCP-1, and TNF-α [15,16], whereas the dysregulation of immune mediators in humans has been associated with the secretion of other inflammatory mediators, such as IL-1β, IL-8, CCL2, CCL3, CCL13, CXCL1, CXCL10, CXCL11, CXCL12, IL6, MIF, SPP [17,18,19]. In addition, severe inflammatory cytokines/chemokines may spill over into the circulation and result in systemic cytokine storms, which are responsible for multi-organ dysfunction and for the impairment of the vascular system and disseminated intravascular coagulation [20,21]. Dendritic cells (DCs) play an essential role in the link between the innate and adaptive immune response, and their maturation is essential for the correct functionality of DCs, such as the migration, processing, and presentation of viral antigens to T- and B-cells for their activation and correct viral clearance [22,23]. EBOV infection has been shown to influence these mechanisms through impairment of DCs in upregulating co-stimulatory molecules (CD40, CD86, and CD80) and Major Histocompatibility Complex (MHC) class II, as well as soluble chemokines and cytokines [24]. EBOV infection is also able to influence the adaptive immune response: severe lymphopenia and the destruction of lymphoid tissue is one of the hallmarks of EBOV infection. Fatal cases showed a more marked reduction of NK cells and γδ T-cell frequency, as well as a loss of peripheral blood CD4+ and CD8+ T cells [25,26]. Moreover, a recent study showed that patients with fatal outcome presented lower, or often absent, levels of both EBOV-specific IgM and IgG, which, when detected, appeared later than in survivors [19]. Overall, the alteration of the innate and adaptive response explains the paralysis of the immune system and its inability to initiate and maintain a protective immune response. At the pulmonary level, many of the pathological changes are, in fact, secondary to systemic alterations, correlating with general pathogenic mechanisms, which are the major causes of severe disease in humans, even at the respiratory level [19,27].

## 3. Ebola Virus Disease 

EVD is a viral hemorrhagic fever (VHF) characterized by acute systemic manifestations with vascular damage, plasma leakage, severe inflammation, and disruption of the immune system [28]. EVD transmissibility seems to vary depending on the stage of disease [29]. A high-level of EBOV replication, associated with systemic dissemination to multiple cell types, results in a complex pathogenesis, which is linked to an increased risk of infection transmission [29]. As stated above, these pathogenic mechanisms include detrimental immune suppression and over-activation of the immune response, disordered coagulation, and tissue damage due to direct viral and indirect host-mediated effectors. In the absence of adequate supportive care, these processes commonly result in multiple organ failure and death within about 10 days of symptom onset in humans. 

It is well recognized that EBOV infection is acquired by direct contact with bodily fluids. Notably, studies conducted in animal models have instilled doubts about possible airborne/droplet transmission (see Section 3.1). However, this route of infection in humans is still debated. Piercy and colleagues evaluated the actual stability of the virus particles in aerosol droplets [30]. They created Ebola-containing aerosol droplets and, according to the decay rates, estimated that EBOV and RESTV can survive in aerosols for roughly 100 and 160 min, respectively, at 50% to 55% relative humidity and 22 ± 3 °C [30]. Therefore, a key additional question to ask is whether primary pulmonary infection of EBOV could be a potential scenario for the future. A fair amount of studies, based on animal experiments (Table 1) and clinical evidence collected during the outbreaks (Table 2), suggest that pulmonary infection may be a possibility. This possibility will be fully investigated below.

### 3.1. Animal Studies

After its first discovery in 1989 in cynomolgus macaques imported to Reston, Virginia, RESTV was detected in domestic swine in the Philippines in a co-infection with the Porcine Reproductive and Respiratory Syndrome Virus (PRRSV, family Arteriviridae, genus Arterivirus) and Porcine Circovirus type 2 (PCV-2; family Circoviridae) [32,34]. Later on, RESTV was identified to cause asymptomatic infections with mild respiratory symptoms, which may result in severe mortality in cases of co-infections with other viral pathogens like viruses in the families Arteriviridae and Circoviridae. The virus was first isolated in lung and lymphoid tissues in the original disease investigation [32]. However, the massive presence of the virus in the lungs may be due to the fact that RESTV infection in pigs has been mostly associated with other infections of the respiratory tract, which may contribute to the specific localization of the virus and the respiratory symptoms of the disease. Marsh et al. [33] conducted an experimental study to rule out the effect of other pathogens affecting pigs, using a 2008 Philippines swine isolate of RESTV. Specifically, five-week-old pigs were exposed (via the oro-nasal or subcutaneous route) to the virus, and the subsequent viral replication in internal organs and shedding of the virus from the nasopharynx was observed. The researchers detected the highest levels of virus replication in lung and lymphoid tissues, confirming previous results [32]. 

The detection of RESTV in domestic swine raised important biosecurity concerns about the potential for the disease’s emergence in humans and other livestock, mainly in animals for food consumption [32,33]. The evidence of RESTV seropositive individuals further increased the concern for human infections and the worries of researchers, farm owners, and the public at large (World Health Organization. WHO, 2009, Available online: https://www.who.int/csr/resources/publications/HSE_EPR_2009_2.pdf). Interestingly, so far RESTV has not been seen to result in any human disease, even if there is concern that its passage through swine may allow RESTV to diverge and shift its potential for pathogenicity [58]. 

On the other hand, several studies investigated if other Ebola viruses may be transmitted through the aerosol route and may result in primary pulmonary infection [9,10,59]. Researchers reviewed the different animal models and offered an overview regarding the possibilities of Ebola viruses causing aerosol infections in non-human primates (NHPs) and other animals. Experimental studies analyzed the respiratory tract involvement in filovirus infections when the animals were exposed to the virus through different aerosol routes (artificially aerosolized virus or natural aerosol transmission) [36,37,39,60]. In these experimental studies conducted on NHPs and pigs, EBOV was inoculated via the aerosol route, and, following mucosal exposure, EBOV replicated, reaching high concentrations, mainly in the respiratory tract, with the development of severe lung pathology. Interestingly, Weingartl et al. demonstrated that piglets inoculated oro-nasally with EBOV and then transferred to a different room housing macaques in an open inaccessible cage system resulted in EBOV infection of all macaques, suggesting a need to revise prevention and control measures during outbreaks [37]. Viral replication was observed within alveolar spaces [36,37], in type I pneumocytes and macrophages [36], and in type II pneumocytes, bronchiolar epithelial cells, and endothelial cells [38], supporting the respiratory involvement. The upper and lower respiratory tract, the lymphoid tissues, and the mediastinal lymph nodes showed infection signs, as well [39]. Similarly, in experiments on cynomolgus macaques placed separately in cages with experimentally infected piglets [37], and on guinea pigs exposed via aerosols to a guinea pig-adapted EBOV strain [39], viral antigens were detected within alveolar and septal macrophages, pneumocytes, epithelial cells, endothelial cells, fibroblasts, and other interstitial cells of the respiratory tree [61]. Considering the pathology of the respiratory system, the expression of disease in the lungs and the patterns of lesions seem to be influenced by the exposure routes (aerogenous or hematogenous). Broncho–interstitial pneumonia, characterized by injury to both the bronchiolar and the alveolar epithelium, is commonly caused by aerogenous viral infections [62]. Moreover, such pathological features were generally not evidenced following the inoculation of EBOV by other routes in NHPs and laboratory animals [9,35]. As shown in animal studies, primary pulmonary infections could occur and cause active viral shedding from the respiratory tract, thus potentially setting up a cycle of ongoing respiratory transmission in humans [9,63].

Overall, experimental works conducted so far have shown that EBOV infection induces respiratory complications, that the virus can be shed via the respiratory secretions, and that it can cause similar pulmonary lesions both in animals exposed to aerosols and in those kept nearby in separate cages with no close contact.

### 3.2. Clinical and Pathology Findings in Humans during EVD 

The pathophysiological mechanism of pulmonary disease in patients with EVD is unknown. Notably, autopsies were performed on a limited number of humans (about 30 cases), primarily during the 1976 SUDV and 1995 EBOV EVD outbreaks and revealed interesting characteristics at microscopic level. During the first known SUDV outbreak, chest pain was almost universal (83% of patients), often accompanied by a dry cough. Autopsies were further performed on two patients and thickening of the alveolar walls due to proliferative accumulations of alveolar cells was found [64]. Furthermore, a possible pathogenetic role of the virus in the respiratory tract was suggested by the fact that viral inclusions within alveolar macrophages and free viral particles within alveolar space were found in the lungs from fatal EVD cases who showed congestion, focal intra-alveolar edema, diffuse alveolar damage, and hemorrhaging. [9,10].

One of the most common symptoms in EVD patients is a cough (up to 49%), especially during the progression of the disease, when viral loads in serum significantly increase, and the virus is copiously emitted in most body fluids, as well as in aerosol particles of various sizes [65,66]. Among the reported EVD cases in the literature, respiratory symptoms were commonly reported with a wide range of symptoms, such as a cough (from 3% [67,68] to 60% [69]), dyspnoea or breathless (detected from 0% [70] to 49% [57]), and chest pain (from 7.5% [71] to 98.6% [72]). Moreover, a WHO study on the first 9 months of the epidemic in Western Africa found that nearly 30% (194 out of 665) of the patients experienced coughing and 2.4% (20 of 831) had a bloody cough [73,74]. A study of 27 EBOV-positive patients of the 2013–2016 outbreak in Western Africa, treated in Europe and USA, reported that cough and dyspnoea were present at admission in seven (30%) and five (22%) EVD patients, respectively. At symptom onset, only a cough was reported in one patient. Furthermore, during hospitalization, 14 patients (52%) experienced hypoxemia while they were breathing ambient air, 12 patients (44%) had pulmonary oedema, seven patients (26%) had pneumonia, 39 patients (33%) had respiratory failure, and six patients (22%) had a diagnosis of acute respiratory distress syndrome (ARDS). Of these patients, four patients (15%) received non-invasive mechanical ventilation, and seven patients (26%) received invasive mechanical ventilation [75]. Notably, the first EBOV-positive patient treated in Italy, mechanically ventilated for respiratory insufficiency for 5 days, had high levels of EBOV RNA in the lower respiratory tract secretions. The authors concluded that the absence of other identified respiratory pathogens in broncho-alveolar lavage fluids and aspirates supported the hypothesis of a direct contribution to the lung tissues damage by EBOV. Notably, EBOV RNA was detected in bronchial aspirate fluids when the EBOV RNA concentration in the concomitant blood samples was barely detectable. Furthermore, the blood EBOV RNA concentrations in the previous days were significantly lower than the concentrations detected in the bronchial aspirate samples. These findings suggest that this EBOV infection is unlikely a spillover from the blood compartment, eventually accompanied by delayed clearance. Instead, the most plausible explanation is that the virus actually replicated into the lower respiratory tract [76]. 

In the second EBOV-patient treated in Italy, our group investigated the presence of EBOV genetic material in the lungs and blood during the patient’s treatment and recovery. The patient showed a persistence of EBOV replication markers within the respiratory tract, with a prolonged detection of EBOV viral RNAs (negative and positive sense RNAs: neg-RNA and pos-RNA, respectively), known to be associated with EBOV replication, in the lower respiratory tract for up to five days after the EBOV viral load in blood was already undetectable. These results suggest that EBOV may replicate in the lungs, although it is possible that the lungs simply provided a protective environment that allowed RNA to linger longer than it did in the plasma. Nevertheless, the detection of pos-RNA together with neg-RNA in the sputum (until day 9 and 10 of the hospital stay, respectively) supports the concept of active viral replication within the respiratory tract, rather than plasma spill-over or prolonged RNA stability [14].

**Figure 2:**
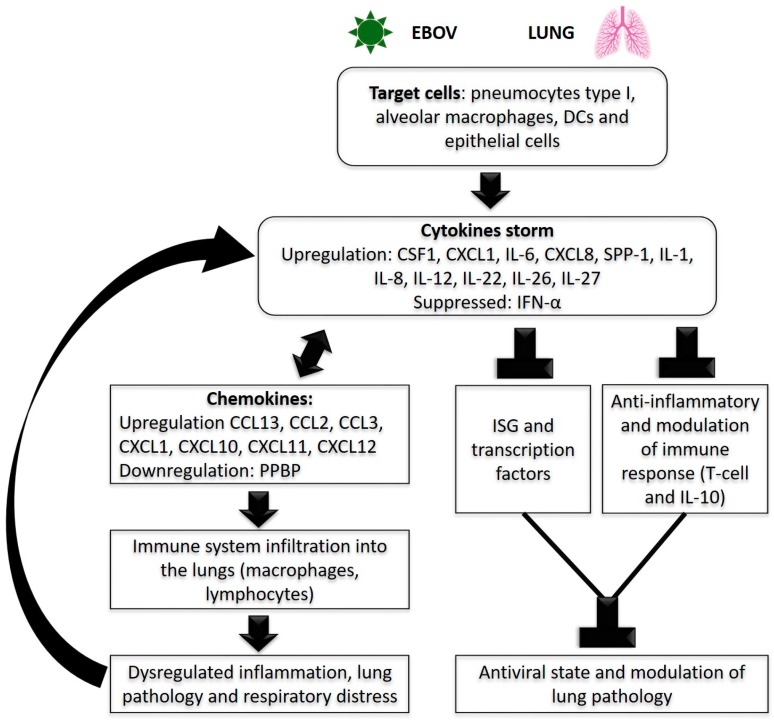
EBOV pulmonary disease pathogenesis. Arrows show the proposed sequence of events and inverted ‘Ts’ show the blocked mechanisms due to the consequences of viral infection.

Overall, the pathophysiological mechanisms of pulmonary disease in patients with EVD are still uncertain, but there could be multiple contributing factors, including vascular leak from endothelial infection, cytokine dysregulation, or direct damage to EBOV-infected cells (Figure 2). 

### 3.3. Impact of Epidemiological and Virological Data on Infection and Control Measures

Our understanding of EBOV transmission in humans mainly relies on epidemiological observations. Contact with bodily fluids from EVD patients remains the most likely route of transmission. Notably, the number of past outbreaks and associated epidemiological studies hat carefully examine transmission patterns is small. Therefore, conclusions about transmission are based on relatively limited data sets [10]. Interestingly, 18 (6.6%) of the 2774 cases in the 1976 SUDV outbreak in Nzara, Sudan, and 55 (17.4%) of the 316 cases during the 1995 EBOV outbreak in Kikwit, DRC, had no direct or physical contact with an infected person or known infected dead body [77,78], thus pointing to other possible routes of transmission, e.g., human to human respiratory tract infection through droplet and aerosols. During the 2013–2016 Western Africa epidemic, more than 890 health care workers (HCW) were infected, with a case fatality rate of 57% [60], whereas during the current 2018–2019 outbreak in DRC, as of 22 July 2019, 140 HCW have been already affected (5.4% of total cases) [6]. 

Currently, full body protection is recommend by WHO and CDC [79,80]. All HCW involved in the care of EVD patients must receive training and demonstrate competency in performing all Ebola related infection control practices and procedures, specifically in proper donning and doffing PPE even if using an N95 mask or a powered air-purifying respirator (PAPR). The risk of infection via inhalation of contaminated aerosols from exposed individuals has not been documented. However, droplets containing EBOV that have become aerosolized (e.g., from coughing sneezing, vomiting, invasive medical or surgical procedures, or surfaces) may have the potential to come into contact with a person’s mucous membrane in their nose or mouth or non-intact skin. Therefore, respiratory protection may be helpful in providing a barrier to help prevent infectious materials from contacting a wearer’s mucous membranes. 

Finally, the epidemiologic and viral evidence of EBOV detection and replication in the respiratory tract raise concerns on the need of strict application of cough etiquette for patients and of droplet and/or respiratory precautions for all HCW involved in the clinical management of EVD suspected and confirmed cases.

## 4. Conclusions

Acute respiratory tract infections (ARTIs) remain a leading cause of mortality, morbidity, and economic loss, and viruses are one of the main causes of such disease. WHO estimates that ARTIs cause nearly four million deaths per year, a rate of more than 60 deaths/100,000 people [81]. The microbial etiology of ARIs is varied, with viruses being the most common cause in humans [82], leading to a high level of awareness and the necessity to develop countermeasures to control them (Table 3).

Filoviruses are not commonly considered to be viruses responsible for ARIs, even if respiratory symptoms may be present as a consequence of diffuse systemic alterations. Interestingly, evidence collected in animal studies, in the epidemiological analysis of transmission chains, and in the most recent Ebola outbreaks suggests that EBOV may be able to cause primary pulmonary infection. This evidence highlights the ability of the virus to be shed in the lung, suggesting a role in lung pathogenesis. Specifically, the relevant proportion of EVD patients without any epidemiologic link to the exposure to contaminated biological samples or fomites, or to any contact with EVD patients; the evidence of respiratory signs and symptoms commonly reported all over the clinical course; the abundance of viral antigens in the lungs in animal necropsies; the prolonged persistence of EBOV detection and replication within the respiratory tract days after undetectable EBOV viral load in plasma; and similar clinical patterns in several other viral respiratory tract infections are all different parameters with consistent evidence of a major role in the pathogenesis of EVD in respiratory tissues [10].

On the other hand, there is no evidence of aerosol transmission in EVD. However, different studies addressing this issue have been performed [30,83], and aerosol transmission was considered a possibility as a consequence of epidemiological observations in past outbreaks, where people showed signs of EVD even in the absence of a direct or physical contact with an infected person or known infected dead body [78,84]. This hypothesis was corroborated by other studies, in which the presence of free viral particles in alveoli and within intra-alveolar macrophages demonstrated a pulmonary involvement [10]. 

From a clinical point of view, the 2013–2016 EBOV outbreak underlined the lung involvement in EVD pathogenesis. In fact, only a few patients treated in Europe and USA had a cough and difficulty breathing at admission. Nevertheless, during the clinical progression, half of the patients experienced hypoxemia while breathing room air, one third had respiratory failure, and one fourth received invasive or non-invasive mechanical ventilation [75]. In the Italian experience at the National Institute for Infectious Diseases “L. Spallanzani” (INMI), respiratory symptoms were present in both patients, in the absence of other common respiratory pathogens [14,76]. One case required mechanical ventilation and the other presented EBOV replication markers in the lungs even after clearance of the virus from the blood. The INMI experience suggests a direct role of the virus in lung pathogenesis.

Although lung pathogenesis in EVD may be secondary to systemic alterations (correlating with general pathogenic mechanisms) the direct presence of the virus is undisputable in the lung, and its interaction with the immune system, whose hyper-activation may be the most likely explanation of the lung damage, is also indisputable. Further research will be needed to better understand the potential role of pulmonary involvement in EVD and whether it may be a factor in the transmission of the virus from one human to another. 

## Figures and Tables

**Table 1:** Evidences in animal studies of Ebolavirus (EBOV) infection and pathogenesis in the respiratory system.

Year	Animal	Virus (Co-Infection; Provenance)	Analyzed Tissue	Route of Inoculation	Pathological Findings	Clinical Signs	Reference
1989	Cynomolgus monkeys (*Macaca fascicularis*)	RESTV (co-infection with SHFV; Philippines)	Plasma, Sera, tissues	Natural infection	Enlarged spleens and kidneys	Anorexia, cough, nasal exudates, swollen eyelids	Jahrling PB, et al., Lancet 1990 [31]
2008	Domestic swine	RESTV (co-infection with PRRSV and PCV-2; Philippines)	Plasma, sera, tissues (lymph nodes)	Natural infection	RESTV isolation from lung and lymph nodes	Severe respiratory disease syndrome, interstitial pneumonia	Barrette RW, et al., Science 2009 [32]
2011	Domestic pigs	RESTV (Philippines swine isolate)	Blood, swabs, tissues	Challenge by subcutaneous and oral routes	RESTV isolation from superficial (submandibular, axillary, inguinal) and internal (bronchial, mesenteric) lymph nodes, nasal turbinates, muscle, and lung	Mild acute rhinitis Acute bronchopneumonia	Marsh GA, et al., JID 2011 [33]
2014	Domestic pigs	RESTV (co-infection with PRRSV, China)	Spleen	Natural infection	Positive for RESTV RT-PCR	Typical clinical signs of porcine reproductive and respiratory syndrome	Pan Y, et al., Arch Virol 2014 [34]
1995	Monkeys (*Macaca mulatta*)	EBOV (EBOV isolate)	Plasma, tissues	Aerosol exposure	Bronchial and bronchiolar epithelium, alveolar pneumocytes, and alveolar macrophages showed positive EBOV antigen staining	Typical signs of EVD. Serosanguineous nasal discharge, subcutaneous haemorrhage	Johnson E et al., Int. J. Exp. Path. 1995 [35]
2010	Domesticated Landrace pigs	EBOV (EBOV strain Kikwit 95)	Blood, tissues	Intranasal, intraocular and oral routes	Macroscopic pathological changes in lungs. Abundant viral antigen detection in lungs	Most prominent and progressive clinical signs were respiratory	Kobinger G, et al., J Infect Dis 2011 [36]
2012	Pigs (breed Landrace) and cynomolgus macaques (*Macaca fascicularis*)	EBOV (EBOV strain Kikwit 95)	Blood, tissues	Oro-nasal inoculation of the pigs. Macaques in close contact with the pigs to evaluate aerosol transmission	Pigs: viral antigens in bronchioles Macaques: EBOV antigen in alveolar, septal macrophages, pneumocytes and endothelial cells	Pigs: broncho-interstitial pneumonia with a lobular pattern Macaques: typical signs of EBOV infection, with damages mainly to the lung and liver	Weingartl HM et al., Sci Rep. 2012 [37]
2013	Domesticated Landrace pigs	EBOV (EBOV strain kikwit 95)	Blood, tissues	Intranasal, intraocular and oral routes	Pneumonia, distributed primarily in the dorso-caudal lobes, characterized by consolidation and haemorrhage affecting more than 70% of the lung tissue	Typical signs of EBOV infection. An increase in respiratory rate as well as difficult, abdominal breathing, inappetence, weakness and reluctance to move	Nfon CK, et al., Plos One 2013 [38]
–	Rhesus macaques (*Macaca mulatta*)	EBOV (EBOV strain from Zaire 95)	Blood, set of tissues from all major organs	Aerosol exposure	Histologic changes within the lungs included alveolar histiocytosis, alveolar fibrin, and multifocal fibrinoid vasculitis	Typical signs of EBOV infection	Twenhalfel NA, et al., Vet Path 2013 [39]

PRRSV—Porcine Reproductive and Respiratory Syndrome Virus, SHFV—Simian Haemorrhagic Fever Virus, PCV-2—Porcine Circovirus Type 2, RESTV—Reston Ebolavirus, EBOV—Ebola virus.

**Table 2:** Evidence of lung involvement from retrospective cohort studies and clinical observations from the field.

Date	Country	Virus	No of Cases	No of Deaths	CFR	Clinical Evidence	Diagnostic Evidence	Reference
Jun–Nov 1976	Sudan	SUDV	284	151	53%	Chest pain 153 (83%), Cough 90 (49%) of 183 patients	2 autoptic findings with proliferative thickening of alveolar septa	WHO. Bull WHO 1978 [40]
Aug 1976	Zaire	EBOV	318	280	88%	Cough 36% in 208 deceased patients, 18% in 34 serogically confirmed patients	Clinical evidence	WHO. Bull WHO 1976 [40]
Jun 1977	Zaire	EBOV	1	1	100%	No respiratory sign	Clinical evidence	Heymann DL. J Infect Dis 1980 [41]
Aug–Sep 1979	Sudan	SUDV	34	22	65%	No respiratory sign	Clinical evidence	Baron RC. Bull WHO 1983 [42]
1989	Philippine	RESTV	3	0	0%	No respiratory sign	Clinical evidence	Miranda ME. Lancet 1991 [43]
1990	USA	RESTV	4	0	0%	No respiratory sign	Clinical evidence	CDC. MMWR 1990 [44]
1994	Cote d’Ivoire	TAIFV	4	0	0%	No respiratory sign	Clinical evidence	Le Guenno B. Lancet 1995 [45]
Dec 1994–Feb 1995	Gabon	EBOV	52	31	60%	No respiratory sign	Clinical evidence	Georges AJ. J Infect Dis 1999 [46]
May–Jul 1995	Zaire	EBOV	315	250	79%	Dyspnea 55 (25%) of 209	Clinical evidence	Khan AS. J Infect Dis 1999 [47]
Jan–Apr 1996	Gabon	EBOV	60	45	75%	No respiratory sign	Clinical evidence	Georges AJ. J Infect Dis 1999 [46]
Jul 1996–Mar 1997	Gabon	EBOV	37	21	57%	No respiratory sign	Clinical evidence	Georges AJ. J Infect Dis 1999 [46]
Oct 2000–Jan 2001	Uganda	SUDV	425	224	53%	No data	Clinical evidence	Okware SI. Trop Med Int Health 2002 [48]
Oct 2001–Jul 2002	Gabon, DRC	EBOV	124	96	77%	Article not available	No data	WHO. Week Epi Rec 2003 [49]
Dec 2002–Apr 2003	DRC	EBOV	143	128	90%	No data	Clinical evidence	Formenty P. Med Trop 2003 [50]
Nov–Dec 2003	DRC	EBOV	35	29	83%	No data	Clinical evidence	WHO. Week Epi Rec 2004 [49]
Apr–Jun 2004	Sudan	SUDV	17	7	41%	Cough in 11 of 13 cases, 85%	Clinical evidence	WHO. Week Epi Rec 2005 [49]
April 2005	DRC	EBOV	12	10	83%	No data	No data	Article not avalaible
Aug–Nov 2007	DRC	EBOV	264	187	71%	No data	No data	WHO. Week Epi Rec 2007 [49]
Dec 2007–Jan 2008	Uganda	BDBV	149	37	25%	No data	No data	MacNeil AJ. Infect dis 2011 [51]
Dec 2008–Feb 2009	DRC	EBOV	32	15	47%	No data	No data	WHO. Glob Aler Resp 2009 [52]
May 2011	Uganda	SUDV	1	1	100%	No respiratory symptoms –Respiratory failure	Clinical evidence	Shoemaker T. EID 2012 [53]
Jun–Aug 2012	Uganda	SUDV	17	7	41%	No data	No data	Albarino CG. Virol 2013 [54]
Jun–Nov 2012	DRC	BDBV	35	13	36%	No data	No data	Albarino CG. Virol 2013 [54]
Dec 2013–Jan 2016	Western Africa	EBOV	28,616	11,310	39%	Cough, dyspnoea, pulmonary oedema, pneumonia	Viral replication markers in sputum samples	Baize S. N Engl J Med 2014 [3]
Aug–Nov 2014	DRC	EBOV	66	49	74%	Difficult breathing 21.4% Cough 18%	Clinical evidence	Maganga GD. N Eng J Med 2014 [55]
May 2017	DRC	EBOV	8	4	50%	Cough 25%	Clinical evidence	Nsio J. J Infect Dis 2019 [56]
May–Jul 2018	DRC	EBOV	54	33	61%	Difficult breathing 34.4%	Clinical evidence	The Ebola Outbreak Epidemiology Team Lancet 2019 [38]
August 2018–ongoing	DRC	EBOV	2620	1762	67%	No data	No data	WHO, 2019 [57]

CFR—Case Fatality Rate, DRC—Democratic Republic of Congo, SUDV—Sudan Ebolavirus, RESTV—Reston Ebolavirus, BDBV—Bundibugyo Ebolavirus, EBOV—Ebola virus, TAIFV—Tai Forest Ebolavirus.

**Table 3:** Pathological findings in viral infections.

Family	Genus	Virus	Pathological Findings	Most Common Symptoms	Ref
*Adenoviridae*	*Mastadenovirus*	Adenovirus	Interstitial and peribronchial infiltration, Acute bronchiolitis, Necrosis, Haloed basophilic inclusions	Common cold, Laryngitis, tracheobronchitis	Khanal S. et al., Biomedicines 2018 [85]
*Herpesviridae*	*Cytomegalovirus*	Cytomegalovirus	Interstitial pneumonitis, Intra-alveolar damage, DAD ^§^, Cytomegaly, Eosinophilic intranuclear Cowdry type-B inclusions	Bronchiolitis, Pneumonia *	Falsey AR et al., Semin Respir Crit Care Med. 2007 [86], Pierangeli A Minerva Pediatr. 2018 [87]
*Paramyxoviridae*	*Pneumovirus*	Respiratory Syncytial Virus	Atelectasis, Mucosal ulcerations, DAD Giant cells pneumonia	Common cold, Bronchiolitis ^°,^*, Pneumonia ^°,^*	Pierangeli A Minerva Pediatr. 2018 [87]
	*Morbillivirus*	Measles	Squamous metaplasia of bronchial epithelium, DAD, Multinucleated giant cells	Fever, Sore throat, Tracheobronchitis, Laryngitis	Yanagi Y J Gen Virol. 2006 [88]
*Orthomyxoviridae*	*Influenza*	Influenza virus	Tracheobronchitis, Bronchiolitis, DAD, Hemorrhage oedema, Squamous metaplasia of bronchial epithelium	Fever, Laryngitis, Tracheobronchitis	Capelozzi VL Clinics (Sao Paulo). 2010 [89]
*Coronoviridae*	*Betacoronavirus*	Severe Acute Respiratory Syndrome (SARS)	DAD, Bronchiolar injury, Multinucleated cells Harvey-Comb lung, Acute bronchopneumonia	Bronchitis, Pneumonia	Lau YL Curr Opin Immunol. 2005 [90]
*Flaviviridae*	*Flavivirus*	Yellow fever virus Dengue virus	Alveolar oedema, Interstitila pneumonitis, DAD, DAH ^#^	Pneumonia	Paessler S Annu Rev Pathol. 2013 [91], Lee YR Virus Res. 2007 [92]
*Arenaviridae*	*Arenavirus*	Lassa virus Machupo Virus Guanarito Virus	Alveolar oedema, Interstitial pneumonitis, DAD, Bronchopneumonia	Pneumonia	Paessler S Annu Rev Pathol. 2013 [91], Yun NE Viruses. 2012 [93]
*Buanyaviridae*	*Bunyavirus*	Hantavirus	Alveolar oedema, DAH, Bronchopneumonia	Pneumonia	Paessler S Annu Rev Pathol. 2013 [91], Safronetz D Proc Natl Acad Sci USA. 2014 [94]
	*Nairoviridae*	Crimean-Congo Hemorrhagic Fever (CCHF)	Alveolar oedema	Pneumonia	Paessler S Annu Rev Pathol. 2013 [91]
*Filoviridae*	*Ebolavirus*	Ebola virus	Pneumonia, Pulmonary oedema, Pulomnary effusion	Cough, Bronchitis, Pneumonia	Paessler S Annu Rev Pathol. 2013 [91], Marcinkiewicz J Folia Med Cracov. 2014 [27], Martines RB J Pathol. 2015 [9]
	*Marburgvirus*	Marburgvirus			

^§^ DAD: Diffuse Alveolar Damage, ^#^ DAH: Diffuse Alveolar Hemorrhage, * Immunocompromised patients, ^°^ Most commonly found in infants.
